# ACE: ARIA-CTR Encryption for Low-End Embedded Processors

**DOI:** 10.3390/s20133788

**Published:** 2020-07-06

**Authors:** Hwajeong Seo, Hyeokdong Kwon, Hyunji Kim, Jaehoon Park

**Affiliations:** Division of IT Convergence Engineering, Hansung University, Seoul 02876, Korea; hyeok@hansung.ac.kr (H.K.); 1594012@hansung.ac.kr (H.K.); 20213201@hansung.ac.kr (J.P.)

**Keywords:** ARIA, electronic codebook mode of operation, counter mode of operation, software implementation, embedded processors

## Abstract

In this paper, we present the first optimized implementation of ARIA block cipher on low-end 8-bit Alf and Vegard’s RISC processor (AVR) microcontrollers. To achieve high-speed implementation, primitive operations, including rotation operation, a substitute layer, and a diffusion layer, are carefully optimized for the target low-end embedded processor. The proposed ARIA implementation supports the electronic codebook (ECB) and the counter (CTR) modes of operation. In particular, the CTR mode of operation is further optimized with the pre-computed table of two add-round-key, one substitute layer, and one diffusion layer operations. Finally, the proposed ARIA-CTR implementations on 8-bit AVR microcontrollers achieved 187.1, 216.8, and 246.6 clock cycles per byte for 128-bit, 192-bit, and 256-bit security levels, respectively. Compared with previous reference implementations, the execution timing is improved by 69.8%, 69.6%, and 69.5% for 128-bit, 192-bit, and 256-bit security levels, respectively.

## 1. Introduction

Data encryption is a fundamental technology for secure network communication in the Internet of Things (IoT). However, the data encryption operation imposes high overheads for low-end microcontrollers. For this reason, the efficient implementation of data encryption is important to achieve the high availability of IoT services. Many block cipher algorithms have been suggested by cryptography researchers to achieve this goal.

The international block cipher standard suggested by the National Institute of Standards and Technology (NIST) is the Advanced Encryption Standard (AES) was first introduced in 1998 [1,2]. AES has a 128-bit block size with three different key lengths, including 128-bit, 192-bit, and 256-bit. The basic structure of AES follows that of the Substitution Permutation Network (SPN). Several AES implementations have been introduced for high performance [3,4,5].

The ARIA block cipher was first introduced in 2004 [6]. This block cipher is the South Korea standard (KS X 1213:2004, Korean Standard, Seoul, Korea), IETF standard (RFC 5794), and part of the TLS/SSL protocol. The ARIA block cipher is also based on the SPN structure, and it uses four 8×8 S-boxes for the substitute layer. However, efficient ARIA implementations on low-end microcontrollers have not been achieved.

In this work, we first optimized the ARIA block cipher on low-end embedded processors. Two modes of operation, including the electronic codebook (ECB) and the counter (CTR) operation, are efficiently implemented with optimized rotation operation, a substitute layer, a diffusion layer, and a pre-computed table for repeated data of the initialization vector (IV) in the CTR.

### 1.1. Contribution

#### 1.1.1. First Efficient Implementations of ARIA on Low-End Microcontrollers

Primitive operations for the ARIA block cipher, including a substitute layer, a diffusion layer, and rotation operation, are efficiently implemented on target 8-bit AVR microcontrollers. The proposed method reduces the number of memory accesses and the number of instructions required for primitive operations. Compared with previous implementations, the proposed implementations for key scheduling and encryption optimized the execution timing by 89.1% and 68.0%, respectively.

#### 1.1.2. Optimized ARIA-CTR Encryption with Pre-Computation

The ARIA-CTR mode of operation is further optimized with repeated data of IV. Two add-round-key, one substitute layer, and one diffusion layer are pre-computed in the form of a look-up table (LUT). By accessing the pre-computed table, these expensive operations are efficiently optimized away. ARIA-CTR implementations on 8-bit AVR microcontrollers require 187.1, 216.8, and 246.6 clock cycles per byte for 128-bit, 192-bit, and 256-bit key lengths, respectively.

The remainder of this paper is organized as follows. Section 2 presents an overview of the ARIA block cipher and previous block cipher implementations on 8-bit AVR microcontrollers. In Section 3, the proposed implementations of ARIA-ECB and ARIA-CTR on 8-bit AVR microcontrollers are presented. In Section 4, the performance evaluation of proposed implementation is described. In Section 5, the proposed method is discussed in detail. Finally, conclusions are given in Section 6.

## 2. Related Works

### 2.1. ARIA Block Cipher

A round of the ARIA block cipher consists of three steps, including add-round-key, a substitution layer, and a diffusion layer. The add-round-key performs XOR operation with a 128-bit round key and plaintext. The substitution layer is defined as four types of substitution operations; S-BOX, which are an affine transformation of the inversion function over $GF(2^{8})$. The diffusion layer is a simple linear map operation and performs $GF{\left(2^{8}\right)}^{16}\to GF{\left(2^{8}\right)}^{16}$:

(Y[0],Y[1],⋯,Y[15])→(Z[0],Z[1],⋯,Z[15])

**Figure 1:**
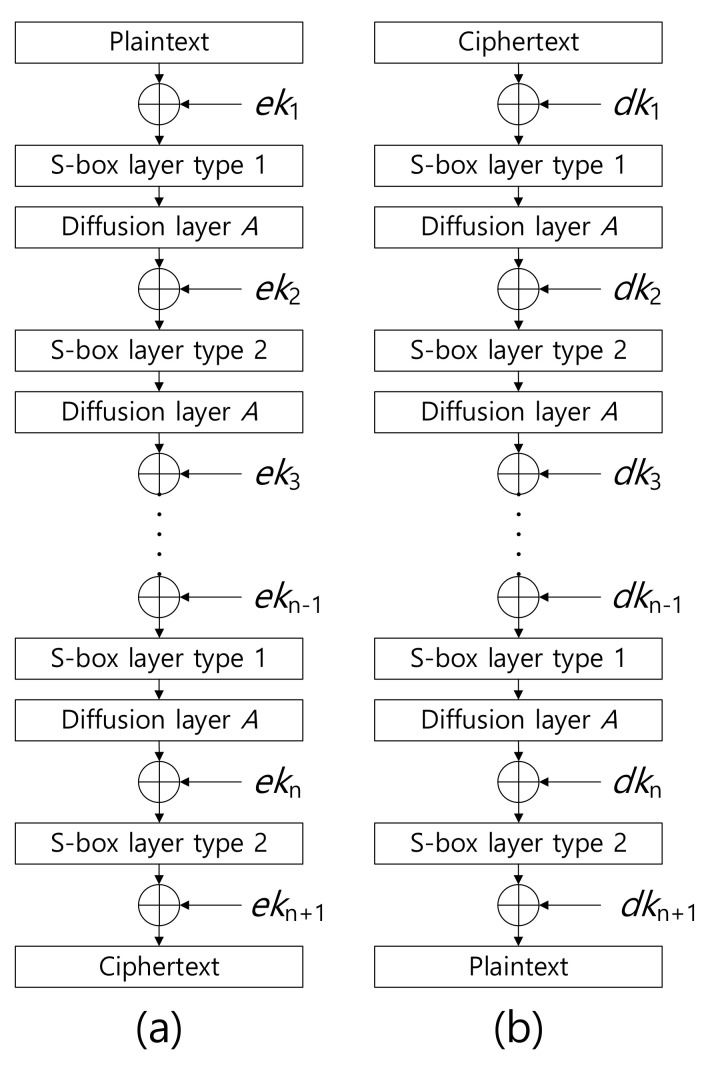
Overview of ARIA (**a**) encryption and (**b**) decryption processes, where ek and dk represent encryption key and decryption key, respectively.

A overview of the ARIA encryption and decryption processes is presented in Figure 1. In particular, encryption and decryption operations have identical architectures. One implementation can support both operations, which optimizes the chip size and code size for hardware and software implementations, respectively.

### 2.2. Block Cipher Mode of Operation

The electronic codebook (ECB) mode is the simplest of the encryption modes. The long message is divided into blocks. Each block is encrypted separately.

An alternative mode of operation is the counter (CTR) mode. The counter mode turns a block cipher into a stream cipher. The CTR mode generates the next keystream block by encrypting successive values of a counter value.

### 2.3. Previous Block Cipher Implementations on 8-Bit AVR Microcontrollers

AVR is a modified Havard architecture 8-bit RISC single-chip microcontroller [7]. AVR microcontrollers find many applications as embedded systems, such as Arduino development boards. The ATmega128 microcontroller supports an 8-bit instruction set, 128 KB FLASH memory, 8 MHz working frequency, two-stage pipeline design, and 4 KB RAM. The number of available registers is 32. Among them, six registers (i.e R26 ∼ R31) are reserved for address pointers, and the remaining registers are used for general purpose registers. The basic arithmetic instruction takes one clock cycle, while the memory access takes two clock cycles per byte. A detailed instruction set summary for implementation is presented in Table 1.

A number of implementation studies have been conducted to improve the performance of block ciphers on 8-bit AVR microcontrollers. Block cipher structures are largely divided into two categories. First, Addition, Rotation, and eXclusive- or (ARX)-based block ciphers have been efficiently implemented on low-end microcontrollers [8,9,10,11,12,13,14,15,16].

In WISA’13, the LEA block cipher was introduced by an institute attached to Electronics and Telecommunications Research Institute (ETRI) [8]. The word size and plaintext size are 32-bit and 128-bit, respectively. Three security levels (128-bit, 192-bit, and 256-bit) are supported. The first implementation of LEA-128 on an 8-bit AVR microcontroller achieved 190 clock cycles per byte for encryption [8]. In WISA’15, speed-optimized and memory-efficient Lightweight Encryption Algorithm (LEA) implementations were presented [9]. The speed-optimized implementation utilizes a byte-wise rotation operation. For the memory-efficient implementation, a partially unrolled approach is used for small code size and reasonable execution timing. In [10], the number of general purpose registers and the instruction set of the AVR microcontroller was fully utilized to optimize the LEA block cipher implementation. The implementation was evaluated on the Fair Evaluation of Lightweight Cryptographic Systems (FELICS) framework. It achieved the best implementation in the first round of the competition. In WISA’18, general purpose registers were efficiently utilized to cache the intermediate results of delta variables during the key scheduling of LEA [11].

In CHES’06, the HIGHT block cipher was introduced [12]. 64-bit plaintext and 128-bit key are supported, and ARX operations are performed in 8-bit wise. The basic implementation of high security and light weight (HIGHT) was first introduced in [13]. The execution timing for encryption and decryption is 2438 and 2520 clock cycles per byte, respectively. In [10], efficient rotation operations were introduced, and they achieved high performance. The result won the second round of FELICS. In [14], speed-optimized and memory-efficient HIGHT implementations were presented. For the speed-optimized implementation, the delta update, F0 function, and F1 function were replaced by an 8-bit aligned LUT. For the memory-efficient implementation, the delta update, F0 function, and F1 function were written in bit-wise operations.

The US National Security Agency (NSA) presented two lightweight block ciphers, namely, SIMON and SPECK [15]. The SIMON and SPECK block ciphers are intended for efficient hardware and software implementations, respectively. They support various block sizes (32-bit, 48-bit, 64-bit, 96-bit, and 128-bit) and various key sizes (64-bit, 72-bit, 96-bit, 128-bit, 144-bit, 192-bit, and 256-bit). RAM-minimizing, high-throughput/low-energy, and flash-minimizing implementations for 8-bit AVR microcontrollers were presented in [16].

Second, Substitution Permutation Network (SPN)-based block ciphers have also been actively investigated. Among them, AES implementations have received considerable attention because the block cipher is an international standard. In [3], the S-box pointer was maintained in the Z address pointer for fast memory access. The mix-column computation was efficiently handled with the conditional branch skip. However, previous implementations have mainly focused on ECB mode of operation. However, the CTR mode of operation is most widely used in practice (e.g., TLS/SSL) [17]. In CHES’18, the compact implementation of AES-CTR (i.e., FACE) was presented [5]. The FACE method takes advantage of repeated data in IV by caching a certain amount of the pre-computed result. However, the implementation method is intended for high-end processors and table updating is frequent during computations. For a resource constrained environment, a lightweight variant of FACE (i.e., FACE-LIGHT) implementation was suggested by [4]. With a newly designed cache table for low-end microcontrollers, implementations of AES-CTR achieved 138, 168, and 199 clock cycles per byte for 128-bit, 192-bit, and 256-bit security levels, respectively.

In this work, we first implemented the ARIA block cipher on low-end 8-bit AVR microcontrollers. Then, the CTR mode of operation for the ARIA block cipher were optimized. By utilizing the repeated IV data and the inner architecture of ARIA, two add-round-key, one substitute layer, and one diffusion layer are replaced with one LUT access.

## 3. Proposed Methods

### 3.1. Efficient Implementation of ARIA-ECB

The ARIA block cipher consists of key scheduling, encryption, and decryption functions. As encryption and decryption operations can be performed in one architecture, only the implementation of encryption operation is required. First, the ARIA-ECB mode of operation is optimized. This is the most basic mode of operation for block ciphers, in which 128-bit plaintext is encrypted with the ARIA encryption in specific security keys (i.e. 128-bit, 192-bit, and 256-bit). The encryption operation outputs 128-bit ciphertext.

#### 3.1.1. Key Scheduling

Key scheduling generates round keys based on the master key. First, the master key is transformed to 128-bit variables ($W_{0}$, $W_{1}$, $W_{2}$, and $W_{3}$) with substitute and diffusion layers. These variables are used to generate round keys with rotation and XOR operations during the key scheduling process. In this section, the primitive operations are described in detail.

Transformed variables ($W_{0}$, $W_{1}$, $W_{2}$, and $W_{3}$) should be maintained throughout the round key generation. However, these 128-bit variables cannot be maintained in registers of 8-bit AVR microcontrollers due to the limited number of general purpose registers. The allocation is presented in detail in Table 2.

Instead of registers, these variables are stored in a STACK memory. To access the STACK pointer, the Z address pointer is set to STACK pointer as follows (STACK pointer is located in the 0x3E3D address.):

INR30,0x3D→INR31,0x3E

After the address setting by adjustment of the Z address pointer, the target address of the STACK memory is accessible.

**Figure 2:**
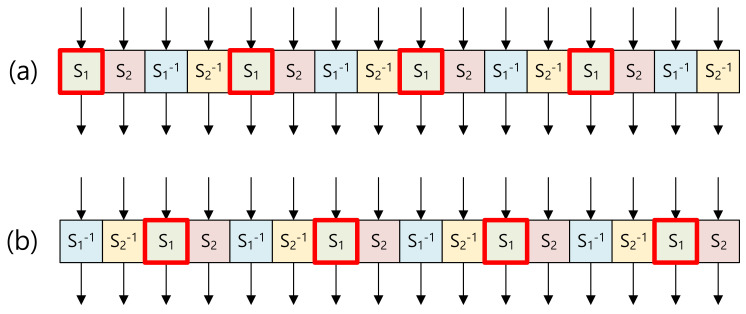
Substitute layer (**a**) type 1 and (**b**) type 2 in a grouped way.

The substitute layer of ARIA consists of sixteen 8-bit-wise S-BOX layers, including four $S_{1}$ layers, four $S_{2}$ layers, four $S_{1}^{-1}$ layers, and four $S_{2}^{-1}$ layers. S-BOX layers type 1 and type 2 are presented in Figure 2. Types 1 and 2 share S-BOX layers, but the order is slightly different. Each S-BOX layer is implemented in the pre-computed table, which receives 8-bit input and generates 8-bit output. To optimize the table access, the memory address is aligned 8-bit wise, where the memory address is 16-bit long for the target microcontroller. With the 8-bit aligned memory address, the lower address is always set to 0x00 value. Only higher 8-bit of address includes the S-BOX starting address. As the offset of the table is 8-bit long, only the lower address must be updated for memory access.

The memory access is performed in a grouped way. In each group, four S-BOX layers are grouped as shown in Figure 2. As an example, the $S_{1}$ box is grouped. Four consecutive memory accesses at the source code level are described in Algorithm 1. In Step 1, the higher address of S-BOX1 (i.e. SBOX1_tbl) is set to the higher address of the Z pointer. In Steps 2–9, four S-BOX1 accesses are performed with input intermediate results (reg1, reg2, reg3, and reg4) by assigning them to the lower address of the Z pointer (R30). Afterward, results are loaded (i.e., LPM instruction) from the FLASH memory to input registers (reg1, reg2, reg3, and reg4).
**Algorithm 1** Optimized four S-BOX1 accesses in a source code level.**Input:** Higher address of S-BOX1 SBOX1_tbl, intermediate results (reg1, reg2, reg3, reg4).
4: MOV R30, reg2**Output:** Output results (reg1, reg2, reg3, reg4)5: LPM reg2, Z1: LDI R31, hi8(SBOX1_tbl)6: MOV R30, reg2: MOV R30, reg18: MOV R30, reg43: LPM reg1, 9: LPM reg4, Z

The diffusion layer requires several XOR operations with input variables. Some of these XOR operation duplicate each other. The diffusion layer is optimized in [6] by re-ordering the computation. Detailed descriptions are given in Algorithm 2.

In Step 1, the $T_{1}$ variable is calculated (i.e., Y[3]⊕Y[4]⊕Y[9]⊕Y[14]). Then, the $T_{1}$ variable is XORed with other values to generate outputs (Z[0], Z[5], Z[11], and Z[14]). This approach optimizes 9 XOR operations (Z[0], Z[5], Z[11], and Z[14]) more than the straight-forward approach. Similarly, the remaining computations ($T_{2}$, $T_{3}$, and $T_{4}$) are calculated with the optimized approach. In total, 36 XOR operations are optimized for the diffusion layer.
**Algorithm 2** 8-bit optimized diffusion layer [6].**Input**: Intermediate results (Y[0]∼Y[15]), temporal registers ($T_{1}$, $T_{2}$, $T_{3}$, $T_{4}$)
10:
$Z\left[15\right]=Y\left[1\right]⊕Y\left[4\right]⊕Y\left[10\right]⊕T_{2}$**Output**: Output of diffusion layer(Z[0]∼Z[15]).
11: $T_{3}=Y\left[1\right]⊕Y\left[6\right]⊕Y\left[11\right]⊕Y\left[12\right]$

1: $T_{1}=Y\left[3\right]⊕Y\left[4\right]⊕Y\left[9\right]⊕Y\left[14\right]$
**12:**$Z\left[2\right]=Y\left[4\right]⊕Y\left[10\right]⊕Y\left[15\right]⊕T_{3}$**2:**$Z\left[0\right]=Y\left[6\right]⊕Y\left[8\right]⊕Y\left[13\right]⊕T_{1}$**13:**$Z\left[7\right]=Y\left[3\right]⊕Y\left[8\right]⊕Y\left[13\right]⊕T_{3}$**3:**$Z\left[5\right]=Y\left[1\right]⊕Y\left[10\right]⊕Y\left[15\right]⊕T_{1}$**14:**$Z\left[9\right]=Y\left[0\right]⊕Y\left[5\right]⊕Y\left[14\right]⊕T_{3}$**4:**$Z\left[11\right]=Y\left[2\right]⊕Y\left[7\right]⊕Y\left[12\right]⊕T_{1}$**15:**$Z\left[12\right]=Y\left[2\right]⊕Y\left[7\right]⊕Y\left[9\right]⊕T_{3}$**5:**$Z\left[14\right]=Y\left[0\right]⊕Y\left[5\right]⊕Y\left[11\right]⊕T_{1}$**16:**$T_{4}=Y\left[0\right]⊕Y\left[7\right]⊕Y\left[10\right]⊕Y\left[13\right]$**6:**$T_{2}=Y\left[2\right]⊕Y\left[5\right]⊕Y\left[8\right]⊕Y\left[15\right]$**17:**$Z\left[3\right]=Y\left[5\right]⊕Y\left[11\right]⊕Y\left[14\right]⊕T_{4}$**7:**$Z\left[1\right]=Y\left[7\right]⊕Y\left[9\right]⊕Y\left[12\right]⊕T_{2}$**18:**$Z\left[6\right]=Y\left[2\right]⊕Y\left[9\right]⊕Y\left[12\right]⊕T_{4}$**8:**$Z\left[4\right]=Y\left[0\right]⊕Y\left[11\right]⊕Y\left[14\right]⊕T_{2}$**19:**$Z\left[8\right]=Y\left[1\right]⊕Y\left[4\right]⊕Y\left[15\right]⊕T_{4}$**9:**$Z\left[10\right]=Y\left[3\right]⊕Y\left[6\right]⊕Y\left[13\right]⊕T_{2}$**20:**$Z\left[13\right]=Y\left[3\right]⊕Y\left[6\right]⊕Y\left[8\right]⊕T_{4}$

The 8-bit optimized diffusion layer approach is efficiently implemented on 8-bit AVR microcontrollers. Detailed descriptions are given in Algorithm 3. The process of $T_{1}$ computation is performed in Steps 1 to 20. The duplicated part ($T_{1}$) is first calculated in Steps 1–4, and the result is stored in the TMP1 register. Then, the TMP1 register is XORed with other registers (Z0, Z5, Z11, Z14), and the remaining XOR operations for these registers are also performed. Similarly, the $T_{2}$ computation is performed in Steps 21 to 40. From the $T_{3}$ computation, the result is stored in the STACK memory (i.e., PUSH) rather than the registers. In Steps 49, 54, 59, and 64, intermediate results are pushed to the STACK memory. Similarly, in the $T_{4}$ computation, intermediate results are pushed to the STACK memory in Steps 73, 78, 83, and 88. In Steps 89 to 96, the pushed results are restored from the STACK memory to the registers. In Steps 97 to 104, intermediate results are moved to the output registers for result alignment.
**Algorithm 3** Proposed implementation of 8-bit optimized diffusion layer in a source code level.**Input:** Intermediate results (Y0∼Y15), temporal register (Z0, Z5,
Z11, Z14, Z1, Z4, Z10, Z15, TMP1, TMP2).
**Output:** diffusion layer intermediate results (Y0∼Y15).
// $T_{1}$ computation
2:EOR TMP1, Y43:EOR TMP1, Y94:EOR TMP1, Y145:MOV Z0, TMP16:EOR Z0, Y67:EOR Z0, Y88:EOR Z0, Y139:MOV Z5, TMP110:EOR Z5, Y111:EOR Z5, Y1012:EOR Z5, Y1513:MOV Z11, TMP114:EOR Z11, Y215:EOR Z11, Y716:EOR Z11, Y1217:MOV Z14, TMP118:EOR Z14, Y019:EOR Z14, Y520:EOR Z14, Y11
// $T_{2}$ computation
21:MOV TMP1, Y222:EOR TMP1, Y523:EOR TMP1, Y824:EOR TMP1, Y1525:MOV Z1, TMP126:EOR Z1, Y727:EOR Z1, Y928: EOR Z1, Y1229: MOV Z4, TMP130:EOR Z4, Y031:EOR Z4, Y11
32:EOR Z4, Y1433:MOV Z10, TMP134:EOR Z10, Y335:EOR Z10, Y636:EOR Z10, Y1337:MOV Z15, TMP138:EOR Z15, Y139: EOR Z15, Y440:EOR Z15, Y10 // $T_{3}$ computation 41:MOV TMP1, Y142:EOR TMP1, Y643:EOR TMP1, Y1144:EOR TMP1, Y1245:EOR TMP1, Y1246:EOR TMP2, Y447:EOR TMP2, Y1048:EOR TMP2, Y1549:PUSH TMP250:MOV TMP2, TMP151:EOR TMP2, Y352:EOR TMP2, Y853:EOR TMP2, Y1354:PUSH TMP255:MOV TMP2, TMP156:EOR TMP2, Y057:EOR TMP2, Y558:EOR TMP2, Y1459:PUSH TMP260:MOV TMP2, TMP161:EOR TMP2, Y262:EOR TMP2, Y763:EOR TMP2, Y964:PUSH TMP2 // $T_{4}$ computation 65:MOV TMP1, Y066: EOR TMP1, Y767:EOR TMP1, Y1068:EOR TMP1, Y1369:MOV TMP2, TMP170: EOR TMP2, Y571:EOR TMP2, Y1172:EOR TMP2, Y1473:PUSH TMP274:MOV TMP2, TMP175:EOR TMP2, Y276:EOR TMP2, Y977:EOR TMP2, Y1278:PUSH TMP279:MOV TMP2, TMP180:EOR TMP2, Y181:EOR TMP2, Y482:EOR TMP2, Y1583:PUSH TMP284:MOV TMP2, TMP185:OR TMP2, Y386:EOR TMP2, Y687:EOR TMP2, Y888: PUSH TMP2 //Finalization 89:POP Y1390:POP Y891:POP Y692:POP Y393:POP Y1294:POP Y995:POP Y796:POP Y297:POP Y298:MOV Y5, Z599:MOV Y11, Z11100:MOV Y14, Z14101:MOV Y1, Z1102:MOV Y4, Z4103:MOV Y10, Z10104:MOV Y15, Z15

The ARIA block cipher requires 128-bit wise rotation operation. Multi-precision rotation on 128-bit wise data (*x*) is efficiently implemented on 8-bit AVR microcontrollers. First, the offset for multiple of 8-bit is performed byte-wise rather than bit-wise. Then, the remaining offset is performed bit-wise. The ARIA block cipher requires five different rotation operations. The 8-bit optimized rotation operation is as follows.

- x⋙19→(x⋙16)⋙3
- x⋙31→(x⋙32)⋘1
- x⋙67→(x⋙64)⋙3
- x⋙97→(x⋙96)⋙1
- x⋙109→(x⋙112)⋘3

Taking an example of 19-bit right rotation, 2-byte is right rotated first and then 3-bit is right rotated. Efficient 1-bit right rotation for 128-bit data is given in Algorithm 4. In Step 1, the most significant bit is cached. Afterward, 1-bit is shifted from the least significant byte to the most significant byte. In Step 18, the least significant bit is replaced with the cached bit from reg16.
**Algorithm 4**ROR_1: 1-bit right rotation for 128-bit data.**Input:** Intermediate results (reg1∼reg16)
**Output:** 1-bit right rotated intermediate results (reg1∼reg16)
1:BST reg16, 02:LSR reg13:ROR reg24:ROR reg3
5:ROR reg46:ROR reg57:ROR reg68:ROR reg79:ROR reg810:ROR reg911:ROR reg1012:ROR reg1113:ROR reg1214:ROR reg1315:ROR reg1416:ROR reg1517:ROR reg1618:BLD reg1, 7

The process of 19-bit right rotation for 128-bit data is given in Algorithm 5. First, 16-bit wise (i.e., 2 bytes) right rotation is performed with the MOVW instruction, which ensures 2-byte-wise register copying. In Steps 10 to 12, the remaining 3-bit right rotation is performed with Algorithm 4 (i.e., 1-bit right rotation for 128-bit data) by calling 3 times.
**Algorithm 5**ROR_19: 19-bit right rotation for 128-bit data.**Input:** Intermediate results (reg1∼reg16), temporal registers (tmp_reg1)
**Output:** 19-bit right rotated intermediate results (reg1∼reg16).
1:MOVW tmp_reg1, reg152:MOVW reg15, reg13
3:MOVW reg13, reg114:MOVW reg11, reg95:MOVW reg9, reg76:MOVW reg7, reg57:MOVW reg5, reg38:MOVW reg3, reg19:MOVW reg1, tmp_reg110:ROR_1 reg1, …, reg1611:ROR_1 reg1, …, reg1612:ROR_1 reg1, …, reg16

Efficient 1-bit left rotation for 128-bit data is given in Algorithm 6. In Step 1, one register is initialized. Then, 1-bit is shifted to the left from the most significant byte to the least significant byte. In Step 18, the most significant bit is replaced by the carry bit generated from Step 17.
**Algorithm 6**ROL_1: 1-bit left rotation for 128-bit data.**Input:** Intermediate results (reg1∼reg16), temporal register (tmp_reg).
**Output:** 1-bit left rotated intermediate results (reg1∼reg16).
1:CLR tmp_reg2:LSL reg163:ROL reg15
4:ROL reg145:ROL reg136:ROL reg127:ROL reg118:ROL reg109:ROL reg910:ROL reg811:ROL reg712:ROL reg613:ROL reg514:ROL reg415:ROL reg316:ROL reg217:ROL reg118:ADC reg16, tmp_reg

The process of 31-bit right rotation for 128-bit data is given in Algorithm 7. First, 32-bit wise (i.e., 4 bytes) right rotation is performed with the MOVW instruction. In Step 11, the remaining 1-bit right rotation is performed with Algorithm 6.
**Algorithm 7**ROR_31: 31-bit right rotation for 128-bit data.**Input:** Intermediate results (reg1∼reg16), temporal registers (tmp_reg1).
**Output:** 31-bit right rotated intermediate results (reg1∼reg16).
1:MOVW tmp_reg3, reg152:MOVW tmp_reg1, reg13
3:MOVW reg15, reg114:MOVW reg13, reg95:MOVW reg11, reg76:MOVW reg9, reg57:MOVW reg7, reg38:MOVW reg5, reg19:MOVW reg3, tmp_reg310:MOVW reg1, tmp_reg111:ROL_1 reg1, …, reg16, tmp_reg1

#### 3.1.2. Encryption & Decryption

As shown in Figure 1, the round function of the ARIA block cipher consists of add-round key, substitute layer, and diffusion layer operations. The add-round key is a simple XOR operation. Substitute layer and diffusion layer operations can be implemented with the method described in the previous section. Details of register utilization for encryption are provided in Table 3. All registers are utilized for high performance.

### 3.2. ACE: ARIA-CTR Encryption for Low-End Processors

In this section, efficient implementations of ARIA-CTR encryption for low-end processors are proposed. The main idea is caching the primitive operations of the ARIA block cipher; this approach skips the operations by the add-round-key of round 2.

#### 3.2.1. Add Round Key

**Figure 3:**
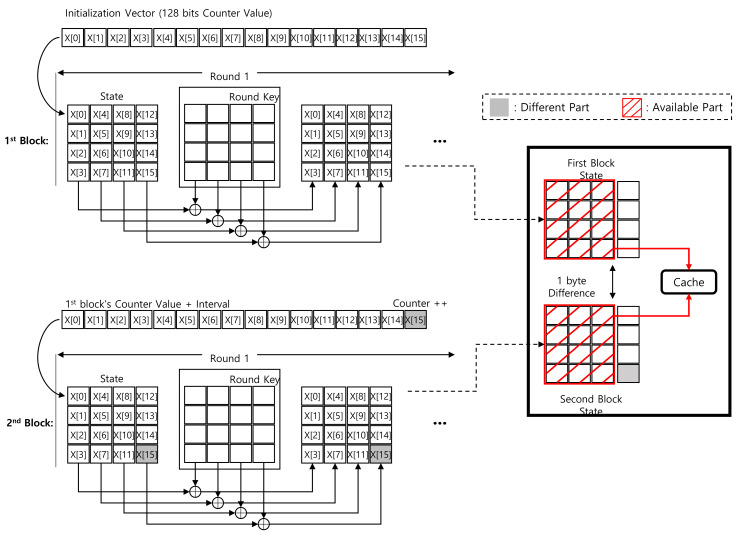
Difference between first and second blocks in Round 1 add-round-key of ARIA-CTR mode of operation.

The first operation of the ARIA block cipher is add-round-key. This is a byte-wise XOR operation with plaintext and round keys. In particular, the CTR mode of operation assigns a (non-constant) 32-bit counter and a (constant) 96-bit IV. Between the first and second blocks, only counter 1 is different in the 32-bit counter section. After the add round key operation, this difference is maintained because it only performs XOR operations. By exploiting this condition, the output of the add-round-key operation can be cached except the counter parts. Detailed descriptions are given in Figure 3. Only the S[15] byte is different between the first and second blocks.

#### 3.2.2. Substitution and Diffusion Layers

**Figure 4:**
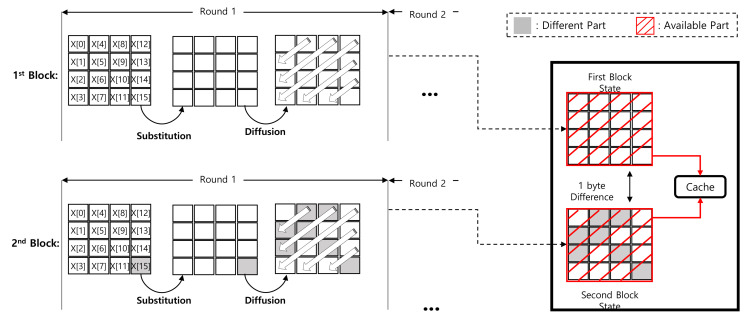
Difference between first and second blocks in Round 1 of ARIA-CTR mode of operation.

The cache table is further extended to the add-round-key operation of round 2. The substitution layer only updates the data byte-wise. The other (constant) bytes are maintained and can be cached. This is presented in detail in Figure 4. The red blocks and gray blocks represent the cached part and other part, respectively.

However, for the diffusion layer, one byte updates other bytes. Taking an example of Y[15], the diffusion layer updates 7 bytes (i.e., Z[1],Z[2],Z[4],Z[5],Z[8],Z[10], and Z[15]) as follows.

$$\begin{array}{c} Z[1]\leftarrow Y[2]⊕Y[5]⊕Y[7]⊕Y[8]⊕Y[9]⊕Y[12]⊕Y[15],\\ Z[2]\leftarrow Y[1]⊕Y[4]⊕Y[6]⊕Y[10]⊕Y[11]⊕Y[12]⊕Y[15],\\ Z[4]\leftarrow Y[0]⊕Y[2]⊕Y[5]⊕Y[8]⊕Y[11]⊕Y[14]⊕Y[15],\\ Z[5]\leftarrow Y[1]⊕Y[3]⊕Y[4]⊕Y[9]⊕Y[10]⊕Y[14]⊕Y[15],\\ Z[8]\leftarrow Y[0]⊕Y[1]⊕Y[4]⊕Y[7]⊕Y[10]⊕Y[13]⊕Y[15],\\ Z[10]\leftarrow Y[2]⊕Y[3]⊕Y[5]⊕Y[6]⊕Y[8]⊕Y[13]⊕Y[15],\\ Z[15]\leftarrow Y[1]⊕Y[2]⊕Y[4]⊕Y[5]⊕Y[8]⊕Y[10]⊕Y[15]. \end{array}$$

**Figure 5:**
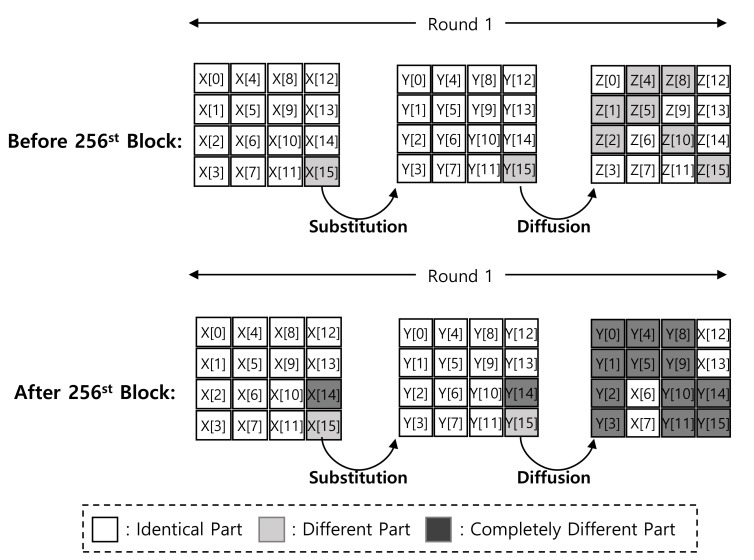
Difference between first and second blocks in Round 1 of ARIA-CTR mode of operation after 256th block.

After the 256-th block, 2 bytes (X[14] and X[15]) are updated, which updates 12 bytes as shown in Figure 5. The counter is 4 bytes, which finally updates all data. For this reason, the straightforward LUT approach does not cache the result, correctly.

To resolve this issue, two pre-computed tables are utilized. The method consists of offline table construction and online computation.

First, the offline LUT construction is performed according to Algorithm 8. In Steps 1–5, add-round-key and substitute layer operations are computed with the constant IV part of plaintext (i.e. X[0]∼X[11]).

In Steps 6 to 21, the diffusion layer is computed with the output of previous steps (Y[0],⋯,Y[11]). While the diffusion layer operation is performed, the second round key ($R_{2}$) is added to the intermediate result. After the computation, these 16 bytes results are cached in a table (Z[0],⋯,Z[15]).

In Steps 22 to 26, new S-BOX tables are constructed. All possible 8-bit values (0∼255) and round keys are XORed and used for the input of substitute layers. The ARIA block cipher requires four different S-BOX tables, which requires 1 KB (256×4).
**Algorithm 8** Offline: LUT computations for ARIA-CTR.**Input:** Plaintext (X[0]∼X[11]), First round key ($R_{1}\left[0\right]∼R_{1}\left[15\right]$), Second round key ($R_{2}\left[0\right]∼R_{2}\left[15\right]$).
**Output:**
Pre-computed diffusion layer (Z[0]∼Z[15]), Pre-computed S-BOX ($NEW\_S_{1},NEW\_S_{2},NEW\_S_{1}^{-1},NEW\_S_{2}^{-1}$).
1:**for** i = 0 to 3 **do**2:$Y\left[i\times 4+0\right]\leftarrow S_{1}\left[X\left[i\times 4+0\right]⊕R_{1}\left[i\times 4+0\right]\right]$3:$Y\left[i\times 4+1\right]\leftarrow S_{2}\left[X\left[i\times 4+1\right]⊕R_{1}\left[i\times 4+1\right]\right]$4:$Y\left[i\times 4+2\right]\leftarrow S_{1}^{-1}\left[X\left[i\times 4+2\right]⊕R_{1}\left[i\times 4+2\right]\right]$5:$Y\left[i\times 4+3\right]\leftarrow S_{2}^{-1}\left[X\left[i\times 4+3\right]⊕R_{1}\left[i\times 4+3\right]\right]$6:**end for**7:$Z\left[0\right]\leftarrow Y\left[3\right]⊕Y\left[4\right]⊕Y\left[6\right]⊕Y\left[8\right]⊕Y\left[9\right]⊕R_{2}\left[0\right]$8:$Z\left[1\right]\leftarrow Y\left[2\right]⊕Y\left[5\right]⊕Y\left[7\right]⊕Y\left[8\right]⊕Y\left[9\right]⊕R_{2}\left[1\right]$9:$Z\left[2\right]\leftarrow Y\left[1\right]⊕Y\left[4\right]⊕Y\left[6\right]⊕Y\left[10\right]⊕Y\left[11\right]⊕R_{2}\left[2\right]$10:$Z\left[3\right]\leftarrow Y\left[0\right]⊕Y\left[5\right]⊕Y\left[7\right]⊕Y\left[10\right]⊕Y\left[11\right]⊕R_{2}\left[3\right]$11:$Z\left[4\right]\leftarrow Y\left[0\right]⊕Y\left[2\right]⊕Y\left[5\right]⊕Y\left[8\right]⊕Y\left[11\right]⊕R_{2}\left[4\right]$12:$Z\left[5\right]\leftarrow Y\left[1\right]⊕Y\left[3\right]⊕Y\left[4\right]⊕Y\left[9\right]⊕Y\left[10\right]⊕R_{2}\left[5\right]$
13:$Z\left[6\right]\leftarrow Y\left[0\right]⊕Y\left[2\right]⊕Y\left[7\right]⊕Y\left[9\right]⊕Y\left[10\right]⊕R_{2}\left[6\right]$14:$Z\left[7\right]\leftarrow Y\left[1\right]⊕Y\left[3\right]⊕Y\left[6\right]⊕Y\left[8\right]⊕Y\left[11\right]⊕R_{2}\left[7\right]$15:$Z\left[8\right]\leftarrow Y\left[0\right]⊕Y\left[1\right]⊕Y\left[4\right]⊕Y\left[7\right]⊕Y\left[10\right]⊕R_{2}\left[8\right]$16:$Z\left[9\right]\leftarrow Y\left[0\right]⊕Y\left[1\right]⊕Y\left[5\right]⊕Y\left[6\right]⊕Y\left[11\right]⊕R_{2}\left[9\right]$17:$Z\left[10\right]\leftarrow Y\left[2\right]⊕Y\left[3\right]⊕Y\left[5\right]⊕Y\left[6\right]⊕Y\left[8\right]⊕R_{2}\left[10\right]$18:$Z\left[11\right]\leftarrow Y\left[2\right]⊕Y\left[3\right]⊕Y\left[4\right]⊕Y\left[7\right]⊕Y\left[9\right]⊕R_{2}\left[11\right]$19:$Z\left[12\right]\leftarrow Y\left[1\right]⊕Y\left[2\right]⊕Y\left[6\right]⊕Y\left[7\right]⊕Y\left[9\right]⊕Y\left[11\right]⊕R_{2}\left[12\right]$20:$Z\left[13\right]\leftarrow Y\left[0\right]⊕Y\left[3\right]⊕Y\left[6\right]⊕Y\left[7\right]⊕Y\left[8\right]⊕Y\left[10\right]⊕R_{2}\left[13\right]$21:$Z\left[14\right]\leftarrow Y\left[0\right]⊕Y\left[3\right]⊕Y\left[4\right]⊕Y\left[5\right]⊕Y\left[9\right]⊕Y\left[11\right]⊕R_{2}\left[14\right]$22:$Z\left[15\right]\leftarrow Y\left[1\right]⊕Y\left[2\right]⊕Y\left[4\right]⊕Y\left[5\right]⊕Y\left[8\right]⊕Y\left[10\right]⊕R_{2}\left[15\right]$23:**for** i = 0 to 255 **do**24:$NEW\_S_{1}\leftarrow S\left[i⊕R_{1}\left[12\right]\right]$25:$NEW\_S_{2}\leftarrow S\left[i⊕R_{1}\left[13\right]\right]$26:$NEW\_S_{1}^{-1}\leftarrow S\left[i⊕R_{1}\left[14\right]\right]$27:$NEW\_S_{2}^{-1}\leftarrow S\left[i⊕R_{1}\left[15\right]\right]$28:**end for**

After construction of the LUT, both caching tables are used for the ARIA-CTR computation online as described in Algorithm 9. In Steps 1 to 4, new SBOX tables are used to generate the output of the add-round-key and substitute layer operations for counter values. In Steps 5 to 20, the diffusion layer is computed with the pre-computed diffusion layer results (Z[0]∼Z[15]) and previous results (Y[12]∼Y[15]). This approach skips two add-round-key, one substitute layer, and one diffusion layer operations. Then, the general ARIA round function is performed.
**Algorithm 9** Online: LUT based computations for ARIA-CTR.**Input:** Plaintext (X[12]∼X[15]), Pre-computed diffusion layer (Z[0]∼Z[15]), Pre-computed S-BOX ($NEW\_S_{1},NEW\_S_{2},NEW\_S_{1}^{-1},NEW\_S_{2}^{-1}$).
**Output:** Intermediate result (Z[0],⋯,Z[15]).
1:$Y\left[12\right]\leftarrow NEW\_S_{1}\left[X\left[12\right]\right]$2:$Y\left[13\right]\leftarrow NEW\_S_{2}\left[X\left[13\right]\right]$3:$Y\left[14\right]\leftarrow NEW\_S_{1}^{-1}\left[X\left[14\right]\right]$4:$Y\left[15\right]\leftarrow NEW\_S_{2}^{-1}\left[X\left[15\right]\right]$
5:Z[0]←Z[0]⊕Y[13]⊕Y[14]6:Z[1]←Z[1]⊕Y[12]⊕Y[15]7:Z[2]←Z[2]⊕Y[12]⊕Y[15]8:Z[3]←Z[3]⊕Y[13]⊕Y[14]9:Z[4]←Z[4]⊕Y[14]⊕Y[15]10:Z[5]←Z[5]⊕Y[14]⊕Y[15]11:Z[6]←Z[6]⊕Y[12]⊕Y[13]12:Z[7]←Z[7]⊕Y[12]⊕Y[13]13:Z[8]←Z[8]⊕Y[13]⊕Y[15]14:Z[9]←Z[9]⊕Y[12]⊕Y[14]15:Z[10]←Z[10]⊕Y[13]⊕Y[15]16:Z[11]←Z[11]⊕Y[12]⊕Y[14]17:Z[12]←Z[12]⊕Y[12]18:Z[13]←Z[13]⊕Y[13]19:Z[14]←Z[14]⊕Y[14]20:Z[15]←Z[15]⊕Y[15]

## 4. Evaluation

The proposed ARIA implementations were evaluated on a low-end 8-bit ATmega128 microcontoller. The microcontroller supports a 128KB FLASH program memory, 4KB EEPROM, and 4KB SRAM. The performance was measured in terms of code size (byte), RAM (byte), and execution time (clock cycles per byte). The software was implemented over Atmel Studio 7, and the code was compiled in -O2 option. All ARIA implementations are written in assembly language. The function call and variable assignment are written in C language.

In Table 4, details of the performance evaluation of the ARIA block cipher implementations are presented; this includes key scheduling, encryption, and decryption operations.

Previous works saved four S-BOX tables in RAM. Each S-BOX table requires 256-byte [6]. In total, 1 KB of RAM is needed to store all tables. The execution timings of ARIA-ECB-128 for key scheduling, encryption, and decryption are 1967.9, 618.8, and 618.8 clock cycles per byte, respectively. In terms of code size, single ARIA implementation can cover all security levels (128-bit, 192-bit, and 256-bit) with counter updates. Decryption operation can be performed with the encryption implementation. The code sizes for key scheduling, encryption, and total are 2890 bytes, 1942 bytes, and 3406 bytes, respectively.

The execution timings of the proposed ARIA-128-ECB for key scheduling, encryption, and decryption are 214.9, 198.3, and 198.3 clock cycles per byte, respectively. Compared with previous reference implementations, the proposed implementations for key scheduling and encryption optimized the execution timing by 89.1% and 68.0%, respectively [6]. The code size of the proposed ARIA-ECB-128 requires 5938 bytes, 2352 bytes, and 8290 bytes for key scheduling, encryption, and total, respectively. The key scheduling is partially unrolled for high performance, which increases the code size but this is negligible in the target microcontroller. The RAM requirements for key scheduling, encryption, and decryption are 306 bytes, 242 bytes, and 242 bytes, respectively. Unlike previous works, pre-computed S-BOX variables are stored in FLASH memory, which reduces the expensive RAM consumption [6]. Compared with previous works, the proposed implementations for key scheduling and encryption optimized the RAM by 76.4% and 80.6%, respectively [6].

The execution timing of the proposed ARIA-CTR-128 requires 187.1 clock cycles per byte. This result is 5.6% faster than the speed-optimized ECB implementation. Similarly, the implementations of ARIA-CTR-192 and ARIA-CTR-256 require 216.8 and 246.6 clock cycles per byte, respectively. These are faster than ECB implementations by 4.9% and 4.3%, respectively. The code sizes of the speed-optimized ARIA-CTR implementation for key scheduling and encryption are 5938 bytes and 3602 bytes, respectively. Compared with the ECB implementation, the CTR implementation requires 1 KB more for the pre-computed substitute layer and diffusion layer.

## 5. Discussion

In this paper, we presented the optimized implementation of the ARIA block cipher on AVR microcontrollers. Optimization techniques are generally divided into AVR specific optimization and generic optimization. In this section, we describe these optimizations in detail.

### 5.1. AVR Specific Optimization

First, memory access is efficiently performed in a grouped way. The memory address is aligned 8-bit wise, which ensures multiple memory accesses with simple offset modifications. This is described in detail in Algorithm 1.

Second, the 8-bit optimized diffusion layer is presented. The target microcontroller has a limited number of registers. The proposed approach reduces the number of memory accesses by utilizing available registers. This is described in detail in Algorithm 3.

Finally, 5 different rotation operations are optimized for the 8-bit microcontroller. This reduces the offset only below 8-bit wise. This is described in detail in Algorithms 4 and 5.

### 5.2. Generic Optimization

Although the ARIA-CTR encryption (ACE) method is optimized for low-end microcontrollers, the proposed method is a generic algorithm. For this reason, the ACE method can optimize the implementation of ARIA-CTR encryption on other platforms, such as 32-bit ARM and Intel processors, without difficulties. The main idea of the proposed method is pre-computation of the ARIA round function. Because the 96-bit nonce value is constant, a large portion of the round function can be re-used. The pre-computed table skips two add-round-key, one substitute layer, and one diffusion layer operations.

## 6. Conclusions

In this paper, we proposed optimized implementations of ARIA–ECB and ARIA-CTR on low-end 8-bit AVR microcontrollers. The implementation of ARIA–ECB is improved with optimized rotation, substitute layer, and diffusion layer operations. Then, ARIA–CTR implementation is further optimized with two cache tables. This novel approach skips ARIA–CTR computations by the add-round-key operation of Round 2. With these efficient implementation methods, ARIA-CTR implementations on 8-bit AVR microcontrollers require 187.1, 216.8, and 246.6 clock cycles per byte for 128-bit, 192-bit, and 256-bit, respectively.

In future work, the proposed method will be applied to other lightweight block ciphers, such as SIMON and SPECK. Furthermore, we will investigate other microcontrollers to achieve high-speed implementation of the ARIA block cipher.

## Figures and Tables

**Table 1:** Instruction set summary for efficient ARIA implementations on 8-bit AVR microcontrollers.

asm	Operands	Description	Operation	#Clock
ADD	Rd, Rr	Add without Carry	Rd ← Rd+Rr	1
ADC	Rd, Rr	Add with Carry	Rd ← Rd+Rr+C	1
EOR	Rd, Rr	Exclusive OR	Rd ← Rd⊕Rr	1
LSL	Rd	Logical Shift Left	C∣Rd ← Rd<<1	1
LSR	Rd	Logical Shift Right	Rd∣C ← 1>>Rd	1
ROL	Rd	Rotate Left Through Carry	C∣Rd ← Rd<<1∣∣C	1
ROR	Rd	Rotate Right Through Carry	Rd∣C ← C∣∣1>>Rd	1
BST	Rd, b	Bit store from Bit in Reg to T Flag	T ← Rd(b)	1
BLD	Rd, b	Bit load from T Flag to a Bit in Reg	Rd(b) ← T	1
MOV	Rd, Rr	Copy Register	Rd ← Rr	1
MOVW	Rd, Rr	Copy Register Word	Rd+1:Rd ← Rr+1:Rr	1
LDI	Rd, K	Load Immediate	Rd ← K	1
LD	Rd, X	Load Indirect	Rd ← (X)	2
LPM	Rd, Z	Load Program Memory	Rd ← (Z)	3
ST	Z, Rr	Store Indirect	(Z) ← Rr	2
PUSH	Rr	Push Register on Stack	STACK ← Rr	2
POP	Rd	Pop Register from Stack	Rd ← STACK	2

**Table 2:** Register utilization for key scheduling.

Description	Number of Registers
STACK pointer	2
Round key address pointer	2
Intermediate results pointer	16
Temporal registers	10

**Table 3:** Register utilization for encryption.

Description	Number of Registers
STACK pointer	2
Round key address pointer	2
Intermediate results pointer	16
Temporal registers	10
Loop counter	1
Zero constant	1

**Table 4:** Comparison results of ARIA block ciphers on 8-bit AVR microcontrollers in terms of code size (byte), RAM (byte), and execution time (clock cycles/byte), ${}^{1}$: speed-optimized ECB implementation, ${}^{2}$: speed-optimized CTR implementation. EKS, ENC, DEC, and SUM represent encryption key scheduling, encryption, decryption, and summation, respectively.

	Code Size				RAM			Execution Time		
Impl.	(Bytes)				(Bytes)			(Cycles per Byte)		
	EKS	ENC	DEC	SUM	EKS	ENC	DEC	EKS	ENC	DEC
ARIA-128										
Kwon et al. [6]	2890	1942	-	3406	1296	1248	1248	1967.9	618.8	618.8
This work ${}^{1}$	5938	2352	-	8290	306	242	242	214.9	198.3	198.3
This work ${}^{2}$	5938	3538	-	9476	306	242	-	214.9	187.1	-
ARIA-192										
Kwon et al. [6]	2890	1942	-	3406	1336	1280	1,280	1494.7	713.3	713.3
This work ${}^{1}$	6194	2352	-	8546	346	274	274	158.0	228.0	228.0
This work ${}^{2}$	6194	3538	-	9732	346	274	-	158.0	216.8	-
ARIA-256										
Kwon et al. [6]	2890	1942	-	3406	1376	1312	1312	1260.3	807.9	807.9
This work ${}^{1}$	6706	2352	-	9058	386	306	306	130.1	257.8	257.8
This work ${}^{2}$	6706	3538	-	10,244	386	306	-	130.1	246.6	-
